# Connecting the science and practice of implementation – applying the lens of context to inform study design in implementation research

**DOI:** 10.3389/frhs.2023.1162762

**Published:** 2023-07-07

**Authors:** Gillian Harvey, Jo Rycroft-Malone, Kate Seers, Paul Wilson, Christine Cassidy, Mark Embrett, Jiale Hu, Mark Pearson, Sonia Semenic, Junqiang Zhao, Ian D. Graham

**Affiliations:** ^1^ Caring Futures Institute, Flinders University, Adelaide, SA, Australia; ^2^ Faculty of Health and Medicine, Lancaster University, Lancaster, United Kingdom; ^3^ Warwick Medical School, Faculty of Science, University of Warwick, Coventry, United Kingdom; ^4^ Centre for Primary Care and Health Services Research, University of Manchester, Manchester, United Kingdom; ^5^ School of Nursing, Faculty of Health, Dalhousie University, Halifax, NS, Canada; ^6^ Faculty of Health, Dalhousie University, Halifax, NS, Canada; ^7^ College of Health Professions, Virginia Commonwealth University, Richmond, VA, United States; ^8^ Wolfson Palliative Care Research Centre, Hull York Medical School, Hull, United Kingdom; ^9^ Ingram School of Nursing, Faculty of Medicine and Health Sciences, McGill University, Montreal, QC, Canada; ^10^ Centre for Research on Health and Nursing, Faculty of Health Sciences, University of Ottawa, Ottawa, ON, Canada; ^11^ School of Epidemiology and Public Health, Faculty of Medicine, University of Ottawa, Ottawa, ON, Canada

**Keywords:** implementation research, implementation practice, context, adaptation, study design

## Abstract

The saying “horses for courses” refers to the idea that different people and things possess different skills or qualities that are appropriate in different situations. In this paper, we apply the analogy of “horses for courses” to stimulate a debate about how and why we need to get better at selecting appropriate implementation research methods that take account of the context in which implementation occurs. To ensure that implementation research achieves its intended purpose of enhancing the uptake of research-informed evidence in policy and practice, we start from a position that implementation research should be explicitly connected to implementation practice. Building on our collective experience as implementation researchers, implementation practitioners (users of implementation research), implementation facilitators and implementation educators and subsequent deliberations with an international, inter-disciplinary group involved in practising and studying implementation, we present a discussion paper with practical suggestions that aim to inform more practice-relevant implementation research.

## Introduction

Implementation science has advanced significantly in the last two decades. When the journal Implementation Science launched in 2006, it defined implementation research as “*the scientific study of methods to promote the systematic uptake of research findings and other evidence-based practices into routine practice, and, hence, to improve the quality and effectiveness of health services*” (1, p.1) and subsequent work has advanced theoretical and empirical development in the field. Yet questions remain as to whether implementation science is achieving impact at the level of health systems and population health (2) and if implementation science is in danger of re-creating the type of evidence-practice gap it was intended to address (2–5). In relation to this latter point – the apparent dis-connect between implementation science and implementation practice – critics have challenged the dominant paradigm of implementation research as it is currently conducted, notably a reliance on methodologies that emphasize experimental control and adherence to clearly specified protocols (3, 6). Why is this problematic and what should we be doing to address it? These are questions that we set out to explore with inter-disciplinary colleagues working in the field of implementation research and practice. In exploring these issues, we recognize that views will differ according to the ontological and epistemological positioning of the individuals and teams undertaking implementation research as this will guide the question/s they are seeking to address, and how. Our starting point is essentially a pragmatic one; we believe that implementation science should be useful to and used in practice. Indeed, some authors conceptualize implementation science more broadly than the study of implementation methods, positioning it as a “*connection between two equally important components, implementation research and implementation practice*” (7, p.2). As such, whilst “*implementation research seeks to understand and evaluate approaches used to translate evidence to the real world. Implementation practice seeks to apply and adapt these approaches in different contexts and settings to achieve positive outcomes*” (8, p.238).

This inter-connectedness between implementation research and implementation practice reflects our starting position and a belief that implementation research should generate transferable and applicable knowledge for implementation practice. In turn, this requires responsiveness and changes to modifiable contextual factors that influence implementation. For example, studies of the effectiveness of facilitation as an implementation strategy have shown mixed results (9, 10) and demonstrated that an important contextual factor is the level of support from clinical leaders in the implementation setting. Whilst this can be factored into the design of future research, leaders may change during the conduct of the study, potentially reducing the level of support for the facilitation intervention. This is a modifiable factor, which can either be reported on, or (the alternative option) acted upon, for example, by an additional strategy to engage the new leader and secure greater support. It is this type of more responsive approach to implementation research that the paper is advocating for.

### Context and the complexity of implementation

Although initially conceptualized as a rational, linear process underpinned by traditional biomedical approaches to research translation (11), the complex, iterative and context-dependent nature of implementation is now well recognized (12, 13). This is apparent in the growing interest in applying complexity theory and complex adaptive systems thinking to implementation and implementation science, including attempts to combine different research paradigms to address the complex reality of health systems (13–15). Central to an understanding of complexity is the mediating role of context in presenting barriers and/or enablers of implementation (16–18). Many definitions of context exist in the literature. In this paper we adopt a broad interpretation of context as “*any feature of the circumstances in which an intervention is implemented that may interact with the intervention to produce variation in outcomes*” (19, p.24). As such, contextual factors exist at multiple levels of implementation from individuals and teams, through to organizations and health systems (17, 20). They do not work in isolation but interact in complex ways to impact implementation success. Contextual factors are represented to varying degrees in an array of implementation theories, frameworks, and models (21, 22), which can help to design theory-informed implementation interventions and predict and explain implementation processes and outcomes (23).

### Advances in implementation science

Alongside the growth of implementation theories and frameworks, empirical studies have helped to establish an evidence base on the relative effectiveness of different implementation strategies, including, for example, audit and feedback, education and training, local opinion leaders and computerized reminders (24). Methodological developments are also apparent, particularly the introduction of hybrid trial designs that aim to simultaneously evaluate intervention and implementation effectiveness (25), increased use of pragmatic trial designs, and published guidance on improving the quality of randomized implementation trials (26). However, against this background of the developing science, the evidence-practice gap has remained largely static over the last 20 years. A key study in the US in 1998 indicated that 30%–50% of health care delivery was not in line with best available evidence (27); subsequent studies, published for example, in Europe (2001), Australia (2012 and 2018) and most recently in Canada (2022) reached similar conclusions (28–31) (Table 1). This suggests that a 30%–40% gap between the best available evidence and clinical practice persists, despite the investment that has gone into building the science of implementation. In turn, this could indicate that we are not putting into practice what we know from empirical and theoretical evidence on implementation and that the promise of implementation science is not being realized in terms of improving putative benefits on health systems and health outcomes. That is, we need to put more focus on the implementation of implementation science.

**Table 1:** Studies assessing appropriateness of care against evidence-based recommendations.

Country	Study	Authors	Year of publication	Appropriateness of care
United States of America	How good is the quality of health care in the United States?	Schuster, McGlynn & Brook (27)	1998	Preventive care – 50% Chronic conditions – 60% Acute care – 70%
Netherlands	Implementation of evidence-based guidelines for clinical practice in family medicine	Grol (28)	2001	67%
Australia	CareTrack: assessing the appropriateness of health care delivery in Australia	Runciman, Hunt, Hannaford, et al. (29)	2008	57%
Australia	CareTrack Kids: Quality of Health Care for Children in Australia, 2012–2013	Braithwaite, Hibbert, Jaffe, et al. (30)	2018	59.8%
Canada	Inappropriate use of clinical practices in Canada: a systematic review	Squires, Cho-Young, Aloisio, et al. (31)	2022	70%

### Approaches to studying implementation

Research to derive the evidence base for different implementation strategies has tended to emphasize questions of effectiveness, with a corresponding focus on experimental study designs that seek to control for, rather than respond to, contextual variation. This runs counter to the recognition that implementation is complex, non-linear, and heavily context-dependent, a fact borne out by large robust implementation trials that report null outcomes and demonstrate through embedded process evaluations the contextual variables that contributed to this result (Table 2). Typically, process evaluations are conducted and reported retrospectively to provide an explanatory account of the trial outcomes – *describing rather than responding and adapting* to contextual factors that influence the trajectory of implementation during the study. Furthermore, when considering implementation studies, there are likely to be broader questions of interest than simply the effectiveness of an implementation intervention, including recognized implementation outcomes such as acceptability, appropriateness, affordability, practicability, unintended consequences, equity and feasibility (40, 41). In this paper, we make the case for re-thinking the relationship between implementation research and implementation practice, highlighting the need to become better at working with context throughout the entire research process, from planning to conduct, analysis, interpretation, and dissemination of results, whilst maintaining relevance and rigour at all stages.

**Table 2:** Selected implementation trials with embedded process evaluations and null results.

Study	Year	Authors	Study design	Main outcome findings	Process evaluation findings to explain null trial result
TRACS – A structured training program for caregivers of inpatients after stroke	2014	Clarke, Godfrey, Hawkins, et al. (32)	Pragmatic, 2 arm cluster RCT; 36 UK stroke units	No clinical or statistical improvement at 6 months on primary or secondary outcomes	Contextual factors, including organisational history, team relationships, external policy and service development initiatives influenced the implementation of the caregiver training program in unintended ways
WISE – Implementation of a self-management support approach (WISE) across a health system	2014	Kennedy, Rogers, Chew-Graham, et al. (33)	Pragmatic, 2 arm cluster RCT; 44 UK general practices	No effect on 12-month primary (patient) outcomes	WISE not embedded because of a perceived lack of relevance and fit to the ethos and existing work and need for resources beyond the immediacy of the participating practices
OPERA – Older People's Exercise intervention in Residential and nursing Accommodation	2014	Ellard, Thorogood, Underwood, et al. (34)	Pragmatic, 2 arm cluster RCT; 78 care homes in England	No observed effect on primary or secondary outcomes	OPERA intervention failed to change the prevailing culture that prioritised protecting clients from harm over encouraging activity Overall low attendance at group exercise sessions and those residents most likely to benefit from the intervention were least likely to engage. Low levels of staff training and few home champions for the intervention
TICD – Tailored Implementation in Chronic Diseases (5 tailored programs for chronic conditions in primary care)	2016 2017	Jäger, Steinhäuser, Freund, et al. (35) Wensing (36)	Cluster RCTs of tailored implementation programs in 5 European countries: Netherlands – cardiovascular disease; 34 general practices England – obesity; 28 general practices Norway – depression; 80 municipalities Poland – COPD; 18 general practices Germany – polypharmacy/multimorbidity; 21 general practices	Little overall observed impact on primary or secondary outcomes	Perceived relevance and credibility of practice recommendations Inability to adapt some of the contextual factors encountered, particularly at the outer context level “Determinants, interventions and contextual factors interacted in complex ways, which reduced their impact” (p.3)
FIRE – Facilitating Implementation of Research Evidence	2018	Rycroft-Malone, Seers, Eldh, et al. (37)	Pragmatic, 3 arm cluster RCT in in 4 European countries; 24 nursing homes	No significant differences in the primary outcome of documented compliance with guidance recommendations	Success of intervention implementation related to contextual factors, including fit and alignment, prioritisation and engagement, which determined a facilitator's opportunity to learn over time and enact the role
EPOCH – A multi-component quality improvement intervention to reduce mortality after emergency abdominal surgery	2018	Stephens, Peden, Pearse, et al. (38)	Stepped wedge cluster RCT; 93 UK hospitals	No improvement in primary outcomes, 90-day survival or hospital length of stay	Variable intervention fidelity at hospital level, difficult to engage clinical colleagues Quality improvement leads were attempting to deliver the intervention in challenging contexts with limited time and resources
T^3^ – Triage, Treatment and Transfer of Stroke Patients	2020	McInnes, Dale, Craig, et al. (39)	Pragmatic, 2 arm cluster RCT; 26 emergency departments in Australia	No improvement in 90-day health outcomes of acute stroke patients	The implementation strategy was unable to overcome system and clinician level barriers. Some contextual factors were outside the control of a senior nurse, including low medical engagement, acceptance of supporting evidence and professional boundaries

We engaged in a series of activities to explore these issues further and contribute to the debate on connecting the science and practice of implementation. Our intent is not to promote one research study design over another, but to stimulate debate about the range of research approaches needed to align the science and practice of implementation.

## Connecting the science and practice of implementation: issues, challenges and opportunities

Our central aim was to produce a discussion paper and practical guidance to enable implementation teams to make better decisions about what study designs to apply and when. This started with a roundtable workshop and meetings amongst a small group of the authors (JRM, KS, PW, GH, IG), followed by wider engagement and consultation with an international group of implementation researchers and practitioners.

Our initial activity started with a two-day face-to-face meeting and subsequent virtual meetings to explore the relationship between context and implementation research methods, particularly how implementation research studies could be designed and conducted in a way that was more responsive to context in real-time. From our own experiences of conducting large implementation trials where contextual factors were highly influential (9, 10, 37, 42, 43), we wanted to explore how we could conduct robust research where context was more than a backdrop to the study. Our intent was to examine whether and how context could be addressed in a formative and flexible way throughout an implementation study, rather than the more typical way of considering it at the beginning (e.g., by assessing for likely contextual barriers and enablers) and/or at the end of the research (e.g., analyzing and reflecting on how well the implementation process went). In these initial deliberations, we considered several different issues including strengths and weaknesses of different research designs in terms of attending to and responding to context; the role of theory in connecting implementation science and practice; the role of process/implementation evaluation; and interpretations of fidelity in implementation research.

The output of these initial discussions was used to develop content for an interactive workshop at the 2019 meeting of the international Knowledge Utilization (KU) Colloquium (KU19). Prior to the COVID-19 pandemic this meeting had been held annually since its establishment in 2001 with participants representing implementation researchers, practitioners, and PhD students. Evaluation and research methods in implementation had been a discussion theme at a number of previous meetings of the colloquium. At the 2019 meeting in Montebello, Quebec, Canada, two of the authors (GH and JRM) ran a workshop session for approximately 80 colloquium participants, titled “Refreshing and advancing approaches to evaluation in implementation research”. The objectives of the workshop were presented as an opportunity:

i. For participants to share their experiences of undertaking implementation research and the related challenges and successes.
ii. To engage the community in a discussion about whether and how to refresh our thinking and approaches to evaluation.
iii. To share and discuss ideas about factors that might be usefully considered in the evaluation of implementation interventions.

A short introduction outlined some of the issues for consideration and discussion in relation to taking account of context, adaptation of implementation strategies, summative vs. formative process evaluation and issues of fidelity. Participants were then split into smaller roundtable groups to discuss the following question:

*How could we design more impactful implementation intervention evaluation studies? Consider:*

- *the whole research cycle from planning and design to implementation and evaluation*
- *designs and methods that enable attention to context, adaptability, engagement, and connecting implementation research and practice.*

After a period of discussion, each table nominated a spokesperson to take part in a facilitated feedback discussion, using a goldfish bowl approach. JRM and GH facilitated the feedback process with other workshop participants observing the “goldfish” bowl. Discussion centred on three main themes: the appropriate use and operationalization of theory in implementation research; consideration of a broader range of study designs in implementation research; and building capacity and capability to undertake impactful implementation research. Notes of the discussion were captured and collated into an overall summary (Table 3). At the end of the session, participants were asked to self-nominate if they were interested in forming a working group to further develop the ideas put forward. Twenty-four responded in the affirmative to this invitation.

**Table 3:** Summary of feedback from KU19 fishbowl discussion.

Theme	Discussion points
Theory	• Think about theory toolboxes, rather than rely on the use of single theories or frameworks • Inclusion of theory knowledgeable members on research teams • Operationalise theory with care
Approaches to implementation research	• Engage intended end-users of research throughout • (Better upfront) investment and planning, including greater attention to the ecosystem of implementation • Choose approaches that enable greater attention to incorporating context (e.g., critical and realist approaches, ethnography) • Select approaches that allow flexibility and adaptability (e.g., adaptive trial designs, stepped wedge designs, participatory and realist approaches) • Consider the need to balance flexibility with rigour • Build programs of research and conduct longitudinal studies • Think about how approaches enable scale up and sustainability of the intervention and/or the implementation strategy • View mechanisms more as a dimmer switch than binary
Capacity and capability	• Learn from other disciplines, e.g., team science • Build communities of practice around ways of implementing and methods of evaluation • Pay attention to building up implementation capacity and capability for implementation in study sites • Build the knowledge base and capacity for effective engagement between end-users and researchers throughout the research process

Following the KU19 event, the participants who had expressed an interest in continued involvement, were emailed a short template to complete. The template asked them to list up to 5 key issues they thought should be considered in relation to implementation research that was attentive to context and enabled adaptability, noting why the issue was important and when in the research cycle it was relevant to consider. This feedback was synthesized and fed back in a second round of consultation, giving participants the opportunity to add any further commentary or reflections and asking them to suggest exemplar study designs that could address the issues identified and any benefits and drawbacks of the approach.

## Connecting the science and practice of implementation – a way forward?

Ten participants responded to the first round of consultation (September 2019) and 9 to the second round (February 2020). A diversity of views was expressed in the feedback, however, there was clear support for working with more engaged, flexible, and context-responsive approaches that could bring implementation practice and research closer together. Suggestions of appropriate research designs were put forward, including theoretical and practical issues to be considered. Feedback was analyzed inductively and findings synthesized by the initial core group of authors (JRM, KS, PW, GH, IG) to identify key themes, presented below.

### Engagement with intended users of implementation research

In line with approaches such as co-design, co-production and integrated knowledge translation (44, 45), participants highlighted the importance of engaging with intended users of implementation research, from community members and patients to clinicians, managers, and policy makers. Different groups can play different roles at different times in relation to implementation research. For example, patients, community members, clinicians and decision-makers can generate questions to be addressed by implementation research, clinicians (working with patients and the public) could be expected to apply the research findings in practice, and managers, educators, and policy makers could have a role in enabling, guiding, and supporting implementation. As such, involvement of intended users of the implementation research should be considered throughout the process of research, from identifying the significant priorities for implementation research to ensuring that implementation strategies are relevant, and findings are appropriately disseminated and actioned, thus increasing the likelihood of success and sustainment. The level of involvement can vary along a spectrum, ranging from passive information giving and consultation through to more active involvement and collaboration, with a corresponding shift in power-sharing amongst those involved (46). For the purposes of consistency throughout the paper, we use the term engagement to refer to the more active level of involvement, namely an equal partnership with intended users of implementation research, herewith referred to as implementation practitioners. We recognize that some roles such as clinical academics and embedded researchers may merge the implementation researcher and implementation practitioner roles (47).

### Context responsiveness and flexibility

The need to embrace a wider range of methods to achieve greater engagement, flexibility and context responsiveness was emphasized, recognizing that different approaches have their own strengths and weaknesses in terms of supporting adaptation to context. Several important challenges were highlighted in relation to adopting more flexible methods, such as understanding the complexity of balancing the requirements of fidelity with adaptation of implementation interventions, and the practicalities of operationalizing concepts in complexity theory, particularly when applying it prospectively. Issues of equity, diversity and inclusion were also viewed as important to consider when thinking about all types of implementation research methods and designs, for example, in terms of representative membership of the research team and the potential influence of contextual factors on accessibility and inclusiveness of the implementation strategy.

### Alternative research approaches

Suggestions of alternative methodologies that could enable greater alignment with and consideration of context included participatory research, case study designs, realist evaluation, mixed methods approaches and trial designs such as stepped wedge and adaptive trials. A key point was raised related to the underlying ontological and epistemological position of implementation researchers. Adopting more context-responsive and adaptive approaches to implementation research was seen to align more closely with constructivist or realist ontology with related implications for interpretations of scientific rigour, fidelity, and the role and influence of the researcher. For example, views on whether and how tailoring and adapting interventions to context presents a threat to the rigour of a study varies according to the underlying philosophy adopted by the research team and the choice of research design. The feedback highlighted a need for this to be considered more clearly and explicitly described by implementation researchers.

### Theoretical and practical considerations

The importance of program theory was highlighted, particularly in relation to theorizing the intended change prior to the start of a research study and focusing on theoretical rather than programmatic fidelity of implementation research (48, 49). Alongside methodological and theoretical positioning, a number of more practical considerations were raised, including clarity about thresholds for intervening to adapt the study design and/or implementation strategy and whether and how adaptation should be actively pursued to maintain equity, diversity and inclusion. Other practical issues identified related to how best to define and capture adaptations over time, how to resource detailed, prospective process evaluations that could fully inform and observe adaptations, and the timeframe for evaluation, which was often seen to be insufficient.

The synthesis of feedback from the consultation process informs the subsequent discussion and suggestions for moving the agenda forward.

## Discussion

Much has been learned from studying and applying implementation methods over the last two decades. However, the persistent gap between research evidence and practice indicates a need to get better at connecting implementation research and implementation practice. From an implementation research perspective, this involves thinking differently about what methods are appropriate to use and when. Whilst perceptions of the implementation process have shifted from a rational-linear view to something that is multi-faceted and emergent, it could be argued that some implementation research has become stagnant and ignores or over-simplifies how context influences real-world implementation rather than working flexibly with the inherent complexity of implementation contexts. From our collective deliberation, we propose that implementation research needs to align more closely with the reality of implementation practice, so that it achieves the ultimate aim of improving the delivery of evidence-informed health care and accelerating the resulting impact on health, provider and health system outcomes.

To achieve this alignment requires several actions that embrace engagement between implementation researchers and implementation practitioners (4, 50). These actions also require an appreciation and acceptance of study designs that enable a higher degree of adaptability and responsiveness to context.

### Engagement with intended users of implementation research

Engagement with implementation practitioners should underpin the research process, as exemplified by approaches such as co-design, co-production, and integrated knowledge translation (51, 52). This helps to ensure that the necessary relationships are in place to clearly understand the implementation problems to be addressed, the goals to be achieved, resource and support requirements, and what research methods and adaptations will be required to achieve identified goals. This includes clarity around the implementation outcomes of interest, for example, effectiveness of the implementation strategy; acceptability to key user groups, including patients, consumers and staff; feasibility; and costs of implementation. These are all factors that should be taken into account when selecting an appropriate evaluation study design. It is important to highlight that this approach to engagement is not simply a feature of research approaches such as participatory research but should be a principle underpinning all implementation research studies that aim to improve the uptake of research evidence in practice and policy. It requires particular attention to the relational aspects of implementation, such as fostering local ownership of the problem to be addressed and building capability and capacity amongst both researchers and end-users of research to engage in effective collaboration. Clinical academics and embedded researchers offer one way of bridging the implementation research-practice boundary, including insights into specific contextual factors that could affect implementation processes and outcomes (53).

### Appreciation and acceptance of study designs to enable responsiveness to context

Contextual influences are important at the planning (protocol development), execution and/or analysis phases of an implementation project. Some research designs lend themselves better to engaged approaches with intended end-users of the research to identify, manage and interpret contextual factors. Other than natural experiments, most designs have the potential to consider contextual factors at the protocol development phase, for example by assessing for potential barriers and enablers posed by contextual factors. However, not all study designs present an opportunity to act upon and modify the identified contextual factors in a responsive way. This is particularly the case for experimental studies that are purposefully designed to neutralise context throughout the research process, although more recent developments such as the adaptive trial design offer greater flexibility to account for contextual factors (54). Similarly, all designs present an opportunity to reflect upon contextual influences that affected the outcomes of implementation, particularly if there is a concurrent process evaluation of what is happening during implementation. However, whether this analysis is undertaken prospectively or retrospectively will determine the extent to which the data can inform real-time responsiveness to contextual factors. There is also variability during the execution of the study, as some designs are more amenable to adaptation of the implementation strategy, in response to (often unanticipated) contextual barriers and enablers. Typically, the more responsive or flexible approaches, such as participatory research and quality improvement, have inbuilt feedback loops which allow real-time monitoring, evaluation, and adaptation.

It is interesting to reflect on the effects that the COVID-19 pandemic had in terms of catalysing rapid change in a health system that is known to be slow to transform (55). Flexible approaches to implementation that were responsive to health system needs were critical for enabling rapid change (56). The pandemic response has highlighted the potential for adaptation to context in real-time and contributed to calls for rapid implementation approaches (57). However, rapid approaches to implementation must be considered alongside intentional engagement of end-users. Recent research, which aligns with our anecdotal experience, has shown a decrease in engagement among patients, the health system, and researchers during pandemic planning and response (58); some argue that there is no time to work in true partnership so researchers are falling back into more traditional directive modes of working. In part, this reflects the expectations of research commissioners and policy makers who, drawing on the COVID-19 experience, have a general expectation of more rapid approaches to translation and implementation. While we argue for research designs with higher degrees of adaptability and responsiveness to context, we caution those responsible for conducting and commissioning implementation research not to prioritize speed at the expense of effective collaboration.

### Applying a lens of context to select appropriate research study designs

Building upon the feedback from our iterative discussions and consultation with implementation researchers and practitioners, we have developed a “horses for courses” table of study designs in terms of their potential to respond and adapt to contextual factors at different stages of the research process (Table 4). For each study design, we provide a brief description before indicating when and to what degree it can respond to contextual factors at the protocol development, study execution and/or analysis phase. For each of the three phases, we indicate the potential (high, medium or low) to respond to contextual factors, resulting in an overall high, medium, or low rating (colour coded accordingly in the table). This does not necessarily mean that some approaches are “better” than others, as each needs to be considered in terms of their strengths and weaknesses and the potential trade-offs when selecting one design or another. These considerations are addressed in the final column of the table.

**Table 4:** Summary of selective study designs with potential to respond to context during research phases of protocol development, execution of the study and analysis of findings.

Research design	Description	Responsiveness of study design to context			Considerations	Examples from literature
		Protocol develop-ment	Study execution	Analysis		
Participatory research	Defined by various terms, including participatory action research, community based participatory research, engaged scholarship, and integrated knowledge translation. It involves an approach that “partners the researcher and participants in a collaborative effort to address issues in specific systems” [(15) p.2] and to “to foster democratic processes in the co-creation of knowledge” [(59) p.7]	H	H	H	Engagement with intended end-users is a pre-requisite.	(60, 62)
					Need to consider the time and resources required to build authentic and trusting relationships between research team members (61).	
Realist evaluation	Realist evaluation is a theory-driven approach driven by the question: what works, how, for whom, in what circumstances and to what extent? It involves developing and testing explanatory theory based on context, mechanism, and outcome configurations (CMOcs). These represent hypotheses about how a program works (O) because of the action of some underlying mechanism/s (M) that only function in particular contexts (C) (63, 64). Typically undertaken iteratively to test and refine theoretical propositions over time.	H	H	H	The theory-based approach of realist evaluation aligns with theory informed and informative implementation research and explicitly explores contextual influences on intervention outcomes (65).	(37, 69)
					Development of CMOcs can be challenging (66). There are published examples of applying realist evaluation in implementation research, particularly to conduct process evaluations embedded within randomized controlled trials (37); however process evaluation needs to be conducted prospectively to enable optimal responsiveness to context and engagement with intended users of the research is important to articulate and refine program theory/ies. Whether a realist approach can be incorporated within randomised controlled trials is an area of debate (67, 68).	
Developmental evaluation	Described as an extension of utilization-focused evaluation (70) that is informed by complexity science and systems thinking. The focus is on users and real use of evaluation findings. This involves studying programs in context and understanding program activities as they operate in dynamic environments with complex interactions (71, 72).	H	H	H	Well suited to early stages of implementation and where a need for implementation strategy adaption is anticipated. Does not apply a conventional logic model, but applies systems thinking to map relationships, inter-connections, and assumptions about how change is expected to occur. Researchers need to be comfortable with uncertainty and be willing to change or abandon an intervention and/or implementation strategy mid-course if the data is suggesting another approach might be better. Detailed documentation throughout the study is important to capture decision points and feedback in a timely manner.	(71, 73)
Ethnography	With roots in anthropology, ethnography involves engagement with a small number of study settings to build relationships and undertake in-depth study. Data collection is typically iterative and involves qualitative methods of data collection such as observation, field notes and interviews. As such, if conducted in a participatory way, it is potentially well suited to incorporating end-user perspectives and examining complex implementation processes and contextual influences on implementation (74).	H	H	H	Evidence of increasing use in implementation research, although meanings of ethnography are contested which can make it difficult to evaluate the rigour of the research (74). As with other participatory approaches, reflexivity is an important skill and practice for researchers undertaking ethnographic study, as is awareness of positionality (75).	(76, 77)
Quality/rapid cycle improvement: Single site Multi-site collaborative	Quality improvement (QI) involves a systematic and coordinated approach to solving a problem using specific methods and tools with the aim of bringing about a measurable improvement [(78) p.3]. QI collaboratives involve groups of professionals coming together in real time, either from within an organisation or across multiple organisations, to learn from and motivate each other to improve the quality of health services. Collaboratives often use a structured approach, such as setting targets and undertaking rapid cycles of change (79).	H	H	H	Healthcare staff are likely to have existing knowledge and experience of quality improvement. There are recognized similarities between QI and implementation research and calls to align them more closely (80, 81). However, QI may lack a strong theory and evidence component compared to implementation science. Evidence on the impact of QI collaboratives is mixed, suggesting they “achieve positive – although limited and variable – improvements in processes of care and clinical outcomes” [(82) p.2] There is evidence to suggest that participation in QI collaborative activities may improve problem-solving skills, teamwork and shared leadership (83).	(82, 84)
Case study: Single site Multiple sites	Defined as “an empirical inquiry that investigates a contemporary phenomenon (the“case”) in depth and within its real-world context” [(85) p.18] Typically, they are observational to understand phenomena and their causal mechanisms, including context. However, case study methods can vary from a more positivist to more constructionist focus, which could influence the extent to which they can respond to context (86).	H	M	M	When case study research is conducted using a prospective approach, then it is possible to identify and respond to contextual barriers and enablers during the study. Multi-site and longitudinal case studies (including studies of failure) are useful to capture the dynamics of implementation and build theory (87). However, in the field of implementation science to date, case studies have been described “as a form of *post hoc* process evaluation, to disseminate how the delivery of an intervention is achieved, the mechanisms by which implementation strategies produce change, or how context impacts implementation and related outcomes”[(88) p.2].	(87, 89)
Adaptive randomized controlled trial	Also described as sequential trial designs, adaptive designs allow for staged modifications to key components of the implementation interventions according to pre-specified decision rules. Unlike conventional experimental designs, where the learning typically occurs after the trial is completed, adaptive designs intend for continual learning as the data accumulate, hence the potential to respond to context (90). Examples include the Sequential Multiple Assignment Randomized Trial (SMART) design (54) and the Multiphase Optimization Strategy (MOST) design (91).	M	M	M	Adaptive designs have mostly been conducted in trials of clinical interventions and there are relatively few published examples of adaptive implementation trials. As there is a need for interim data analysis to inform decisions about modification, there is a need for access to rapidly available and measurable outcome data. Temporal trends are also important to consider and can add to the complexity of data analysis (92).	(93, 94)
Stepped wedge randomized controlled trial	Following a baseline period, the implementation intervention is sequentially rolled out to participants. The order of the roll-out sequence is randomized and by the end of the study all participants receive the intervention. “The design is particularly relevant where it is predicted that the intervention will do more good than harm … and/or where, for logistical, practical or financial reasons, it is impossible to deliver the intervention simultaneously to all participants” [(95), p.1]	M	L/M	M	The sequential nature of roll-out means that participants experience different length intervention periods, which can be problematic as those who come in later have a shorter time to implement.	(96, 98)
					Temporal trends can influence the study results and make data analysis more complex (97). If a prospective process evaluation is embedded with the trial, then there could be potential to respond to identified contextual factors during the conduct of the study.	
Hybrid effectiveness-implementation trial	Originally proposed in 2012 as a type of experimental trial design that could combine questions about the effectiveness of an intervention with questions about how best to implement it (25). Three different types of hybrid design were proposed, ranging from a primary focus on testing intervention effectiveness whilst gathering some data about implementation (Type 1), to placing equal weight on testing both the intervention and implementation strategies (Type 2), or primarily testing an implementation strategy and implementation outcomes whilst collecting some information about the intervention (Type 3).	L/M	L	L/M	The hybrid design approach has been widely adopted in the field of implementation science and suggestions put forward for further development or expansion to address context (99). Initially the focus was on testing clinical interventions alongside implementation, although there are many examples of using the approach to evaluate implementation interventions. Ratings are likely to differ from Type 1 to Type 3; the greater the focus on implementation (Type 3), the greater the potential to respond to context if there is an embedded, prospective process evaluation. A recent reflection paper from the original developers of the hybrid design (100) suggests replacing the term ‘design’ with ’study’ to acknowledge that the hybrid approach can be applied more broadly to non-trial research designs. This has the potential to change the level of responsiveness and adaptation to context.	(101, 102)
Pragmatic randomized controlled trial	In contrast to explanatory trials that aim to test the effectiveness of an intervention under optimal conditions, pragmatic trials are designed to evaluate effectiveness under real-world conditions such as the clinical practice setting (103). The PRECIS (The pragmatic explanatory continuum indicator summary tool) and updated PRECIS-2 tool was developed to help researchers design trials along the explanatory to pragmatic continuum taking account of factors such as eligibility criteria, recruitment, setting, flexibility of delivery and adherence (104).	L/M	L.	L	Frequently employed in implementation studies as they place an emphasis on external validity – asking not whether an implementation intervention can work but does it work in routine clinical or health policy contexts (26). This can involve assessment of contextual factors at the study design stage to inform the implementation strategy, although there would not be an active response to contextual factors that emerge during the study.	(105, 106)
					The pragmatic nature of the research is expected to make findings more generalizable; however, what works in one context rarely works exactly the same in another context, raising questions about the degree of generalizability (103).	
Uncontrolled before and after study (pre-post study design)	Involves the measurement of specified outcomes before and after the delivery of the implementation intervention in the same study site or sites.	L/M	L	L	Relatively simple to conduct but cannot necessarily attribute observed changes to the intervention as other factors, including secular trends and unplanned changes, could be at play. Therefore, results have to interpreted with caution - there may be a tendency to over-estimate the effect size of the implementation intervention (107).	(108, 109)
Controlled before and after study	Similar to the pre-post design described above but a control population as similar as possible to the intervention site is identified and data are collected in both groups before and after implementation.	L/M	L	L	Can be difficult to identify a comparable control group and baseline starting points of the intervention and control groups may differ, meaning that some caution is required when interpreting results.	(110, 111)
Interrupted time series	Attempts to detect whether an intervention has an effect that is significantly greater than the underlying secular trends. This involves collecting data related to implementation outcomes at multiple time-points both pre- and post-intervention.	L	L	L	Need to collect sufficient data points, including pre-intervention, to undertake data analysis. This could have implications for the timescale of data collection and can be easier to do if there is access to routine data that can be used for analysis.	(112, 113)
Natural experiment	The research team do not plan or direct the implementation intervention but rather observe outcomes of interest and antecedents in their natural context (114).	L	L	L	Useful for studying implementation occurring a real-world context, but limited potential to respond to contextual factors during the research.	(115, 116)

Informed by our deliberative discussions, there are several pre-conditions to the study designs described in the table that help to optimise the impact of implementation research. These include a starting position that context is an important consideration in implementation research; the relationship between researchers and end-users of research; the need for process evaluation; and the role and contribution of theory.

As noted, we start from an assumed position that context mediates the effects of implementation and, as such, is something that we should work with, rather than seek to control, in implementation research. The ratings assigned to study design in Table 4 are through a lens that “context matters”. If this is a view shared by the implementation research team, then it is important to select a study design that will enable responsiveness and adaptation to context. We recognize that questions of fidelity arise when adapting implementation interventions to context. One way to address this is by specifying the core and adaptable components of the intervention to inform decisions about when tailoring to context is appropriate (117). Additionally, and as noted in the consultation feedback, it is important to consider fidelity alongside the program theory underpinning an implementation strategy. Theoretical fidelity is concerned with achieving the intended mechanisms of action of an intervention, as opposed to fidelity to component parts of the intervention (48, 49). A second condition relates to active engagement between the researcher/s and the intended users of the implementation research. This has important implications for the researchers' role as they can only optimise adaptation to context if they are working in an engaged way to monitor and respond to context in real-time. Thirdly, we highlight the importance of process evaluation in implementation research, in particular process evaluation that is embedded and prospective to capture changes in context that could have implications for implementation success and to inform timely adaptations to the implementation strategy, as well as potential effects the implementation intervention may have on context over time. A final condition relates to the central role of theory and theorising in study design. In line with established guidance on the development and evaluation of complex interventions (23), our starting position is that implementation studies should be informed by theories that are relevant to implementation. Alongside applying theory to guide study design and evaluation, opportunities to move from theory-informed to theory-informative implementation research should be considered, for example, by theorising the dynamic relationships between implementation strategies, implementers and context during data analysis and interpretation (118). Careful documentation within process evaluations of what adaptations occurred, when, how and why can make important contributions to such theorising. The extent to which these conditions are met or not will influence the level of adaptability and responsiveness to context. All the study designs listed in the table have potential to be responsive to context or increase the level of responsiveness in the way they plan and conduct the study and data analysis. So, for example, study designs rated lower in the table could enhance their responsiveness to context by increasing engagement with intended end-users of their research and/or embedding a prospective process evaluation with iterative data analysis in their study.

### How to use the table

As noted, Table 4 is intended to be used when context is seen as an important consideration in implementation research. It is not intended to be prescriptive or a “rule-book” for study design selection as there is no definitive answer to the question “what is the right implementation research design”? Rather it aims to help implementation research teams (including implementation practitioners partnering with researchers) who believe context is important to implementation success to select study designs that will best enable them to identify and then respond to contextual factors during the development, conduct and analysis phases of research. Exactly which study design is appropriate will depend upon several factors including the stage and scale of the research and what trade-offs are acceptable to the research team in terms of strengths and weaknesses of different study designs. For example, if the study is concerned with early-stage development and field testing of an implementation strategy, questions of interest are likely to focus on feasibility, practicability, appropriateness and fit. Here, approaches classified as highly responsive are particularly beneficial to test and refine the implementation strategies in real-time and develop an in-depth understanding of the mechanisms of action and the relationships between mechanisms, context, and outcomes. At a later stage, questions of effectiveness and cost-effectiveness may become more important, in which case an adaptive trial design (coded as medium level) would be relevant as it can enable a continuing (although more limited) responsiveness to contextual factors.

The important point is that research teams should more critically reflect on who they involve as part of their research team and their choice of research design, according to the questions they are attempting to answer and the outcomes they are seeking to achieve (see Box 1). It is also important to note that the designs presented in Table 4 are not exhaustive nor mutually exclusive. Indeed, there are many examples in the literature where different study designs are combined to bring together their relative strengths (15, 67), although this can raise questions about epistemological fit (68). Similarly, there are variations within some of the study designs listed, such as case studies (86) and hybrid studies (100), reflecting different worldviews and approaches within an overarching study design type.

Box 1 **Reflective questions to guide the selection of context-responsive study design in implementation research**

• Who should be at the table to make decisions about the focus of the study, the questions of interest and the planning, conduct, dissemination and evaluation of the implementation research?
• Does our team reflect principles of equity, diversity and inclusion and accessibility?
• What are we aiming to achieve through the implementation research, for example, what are the research questions we are trying to answer?
• What outcomes are the most important to whom and when?
• Do we have a clear program theory or logic and theoretical framing of the study that team members have developed and agreed upon?
• What do we know about the context/s in which we will be implementing the intervention?
• How much contextual variability do we anticipate that could affect implementation outcomes?
• How flexible are we prepared to be in response to modifiable contextual barriers and enablers in order to optimize implementation outcomes?

## Conclusions

To optimise the potential for implementation research to contribute to improving health and health system outcomes, this paper outlines a paradigm shift in how we conceptualise the relationship between implementation research and implementation practice. We argue that implementation research requires the use of study designs with higher degrees of adaptability and responsiveness to context to align more closely with the reality of implementation practice. Such approaches are critical to improve the delivery of evidence-informed health care and positively impact on patient experience, population health, provider experience, and health system outcomes, contributing to health equity and social justice (119). We recognise that the paper raises questions that require ongoing discussion and exploration, such as how best to balance rigour, fidelity and adaptation to context and how to truly address issues of equity, diversity, accessibility and inclusion. Important debates and developments are already underway in these areas [for example, (120–123)] as are ongoing methodological developments in study design that can help to inform future application and refinement of the ideas proposed in this paper.

## Data Availability

The original contributions presented in the study are included in the article, further inquiries can be directed to the corresponding author.
